# From Legislative Harmonization to Real-World Access: A Scoping Review of Pharmaceutical Regulation and Access to Medicines in Romania

**DOI:** 10.3390/healthcare14050688

**Published:** 2026-03-09

**Authors:** Corina Daniela Negrila, Luana-Maria Gherasie, Sebastian Mihai Armean, Petru Armean

**Affiliations:** 1 General Medicine, “Carol Davila” University of Medicine and Pharmacy, 050474 Bucharest, Romania; corina-daniela.negrila@drd.umfcd.ro (C.D.N.); petru.armean@umfcd.ro (P.A.); 2 “Prof. Dr. D. Hociota” Institute of Phonoaudiology and Functional ENT Surgery, 050751 Bucharest, Romania; 3 Department of Pharmacology, Toxicology and Clinical Pharmacology, General Medicine, “Iuliu Hațieganu” University of Medicine and Pharmacy, 400012 Cluj-Napoca, Romania; sebastian.armean@umfcluj.ro; 4 “Prof. Dr. Th. Burghele” Clinical Hospital, 050659 Bucharest, Romania

**Keywords:** pharmaceutical policy, access to medicines, healthcare utilization, aging population, reimbursement and pricing, medicine shortages, European Union, Romania

## Abstract

**Highlights:**

**What are the main findings?**

- Despite extensive alignment with European pharmaceutical legislation, Romania continues to experience severe delays, shortages, and limited reimbursement of innovative medicines;
- Restricted access to medicines is driven by systemic factors, including pricing policies, reimbursement delays, high co-payments, and parallel export.

**What are the implications of the main findings?**

- Limited access to medicines may increase avoidable hospitalizations and healthcare utilization, particularly among aging and vulnerable populations;
- Policy reforms targeting reimbursement timelines, pricing strategies, and patient co-payments are essential to improve health system efficiency and equity.

**Abstract:**

Objectives: This scoping review aimed to map European and Romanian pharmaceutical legislation and policy-related evidence and to examine how legislative harmonization translates into access outcomes in Romania. Eligibility criteria: Legislative documents, institutional reports, market analyses, and peer-reviewed studies addressing pharmaceutical regulation, pricing, reimbursement, and access to medicines (2000–2024). Sources of evidence: EUR-Lex, the Romanian Legislative Portal, PubMed, Scopus, Google Scholar, and institutional sources (European Commission, OECD, WHO, EFPIA, NAMMDR, CNAS). Charting methods: Data were extracted using a standardized charting form and synthesized narratively across thematic domains (regulatory harmonization, pricing and reimbursement, medicine shortages, comparative EU indicators, and health system implications). Results: Fifty sources were included. The mapped evidence consistently identified three dominant patterns: (1) prolonged time-to-availability for centrally authorized medicines, with mean delays exceeding 800 days in Romania compared with approximately 578 days at EU level; (2) limited availability of innovative therapies, particularly in oncology (approximately 20% availability in Romania versus around 50% EU average); and (3) recurrent medicine shortages associated with low-price regulation and parallel export dynamics. Evidence gaps include limited Romania-specific empirical evaluation of the causal effects of individual policy levers (e.g., external reference pricing, reimbursement timelines, clawback mechanisms). Conclusions: Legislative harmonization alone has not ensured equitable or timely access to medicines in Romania. The evidence suggests that national pricing, reimbursement, and supply governance mechanisms mediate the relationship between EU regulation and real-world patient access, highlighting the need for targeted policy reforms and further empirical investigation.

## 1. Introduction

For decades, the European Union (EU) has maintained a comprehensive pharmaceutical regulatory framework to ensure high standards of quality, safety, and efficacy for medicinal products across Member States [1]. Through centralized authorization procedures and harmonized regulatory requirements, EU legislation aims to guarantee that medicines approved at European level meet uniform scientific and safety standards. However, regulatory harmonization at the level of authorization has not eliminated disparities in real-world access to medicines across Member States. Recent developments, including the COVID-19 pandemic and supply disruptions related to the war in Ukraine, did not create these disparities. Instead, they exposed pre-existing structural vulnerabilities in national pricing, reimbursement, and supply governance systems [2,3].

Access to medicines is a multidimensional concept encompassing availability, affordability, quality, and timely reimbursement, as defined by the World Health Organization (WHO) and the Organisation for Economic Co-operation and Development (OECD). Within these frameworks, availability refers to the physical presence of authorized medicines within the health system, affordability reflects the extent to which medicines can be obtained without financial hardship for patients, and timeliness captures the interval between regulatory approval and effective patient access through reimbursement or routine clinical use. Together, these dimensions provide a coherent policy lens for assessing equity and system performance across countries [4,5].

Within this European context, Romania represents a particularly illustrative case. Despite formal transposition of EU pharmaceutical directives into national legislation and participation in the EU regulatory system, Romania continues to experience prolonged reimbursement delays, limited availability of innovative therapies, and recurrent medicine shortages. Comparative European analyses indicate that Romania records among the longest delays in access to reimbursed innovative medicines, with average waiting times exceeding 800 days between European authorization and patient access, compared with approximately 120 days in Germany [6,7,8]. By early 2021, only one-quarter of the new medicines authorized at the European level had been added to the Romanian reimbursement list, leaving roughly 74% of EU-approved drugs unavailable to Romanian patients [9,10]. At the same time, Romania applies a strict external reference pricing policy that results in some of the lowest regulated medicine prices in the EU. This combination of delayed reimbursement and low-price regulation has been associated with restricted market entry, parallel export dynamics, and supply instability [6].

The coexistence of formal legislative harmonization and persistent access disparities raises an important analytical question: how do EU-level authorization standards interact with national pricing, reimbursement, and supply governance mechanisms to shape real-world access outcomes? While Romanian legislation has largely transposed Directive 2001/83/EC and subsequent EU regulations into domestic law, significant gaps remain between legislative intent and functional access performance. The available evidence is dispersed across legislative texts, institutional reports, market analyses, and empirical studies. Integrative syntheses examining how harmonized regulation translates into national-level access outcomes remain limited [11,12].

This scoping review therefore aims to systematically map the European and Romanian pharmaceutical regulatory landscape and to examine how legislative harmonization translates into access outcomes in Romania across the dimensions of timeliness, availability, and affordability. Specifically, the review seeks to: (1) develop a thematic mapping of regulatory and policy mechanisms influencing access; (2) identify and categorize structural barriers related to pricing, reimbursement, health technology assessment (HTA), and supply governance; (3) contextualize Romania’s performance using comparative EU access indicators; and (4) synthesize these findings within a conceptual policy pathway framework linking EU-level regulation to national implementation mechanisms and downstream health system implications [13].

Given the breadth and heterogeneity of the available evidence—including European and national legislation, institutional policy documents, market reports, and peer-reviewed studies—a scoping review methodology was considered the most appropriate approach. Scoping reviews are particularly suited to mapping complex regulatory and policy environments, clarifying key concepts, and identifying systemic evidence gaps when data sources are fragmented and methodologically diverse [10,11]. Beyond regulatory compliance, access to medicines is also closely linked to healthcare utilization and system efficiency, particularly in aging populations with high burdens of chronic disease. By situating Romania within this broader European and demographic context, this review provides a structured analysis of how pharmaceutical legislation translates—or fails to translate—into equitable real-world access. By systematically mapping regulatory frameworks, policy instruments, and reported access indicators within this broader European and demographic context, this approach provides a comprehensive overview of how pharmaceutical legislation translates into real-world access outcomes [14,15] (Table 1).

### Rationale for Scoping Review and Reporting Standards

The available evidence on how EU pharmaceutical regulation translates into real-world access in Romania is heterogeneous and spans legislation, institutional policy documents, market reports, and peer-reviewed empirical studies. Given this breadth and the exploratory nature of our research questions, a scoping review was considered the most appropriate method to map the field, clarify key concepts, and identify evidence gaps rather than to estimate pooled effects. The review is reported in accordance with the PRISMA Extension for Scoping Reviews (PRISMA-ScR) (Supplementary Materials) and the PRISMA 2020 reporting recommendations [16].

- Population: patients and health system stakeholders in Romania.
- Concept: the relationship between EU pharmaceutical regulation/legislative harmonization and access to medicines (timeliness, availability, and affordability), including downstream implications for healthcare utilization.

Context: the EU regulatory framework and its national implementation through Romanian legislation and policy instruments (pricing, reimbursement, HTA, and supply governance).

## 2. Materials and Methods

### 2.1. Study Design

This study was conducted as a scoping review in accordance with the PRISMA Extension for Scoping Reviews (PRISMA-ScR) guidelines [16]. A scoping review methodology was chosen to comprehensively map the existing legislative, regulatory, and policy-related evidence on pharmaceutical regulation and access to medicines in Romania, an area characterized by heterogeneous data sources and limited peer-reviewed literature.

### 2.2. Protocol and Registration

No prospective protocol was registered prior to conducting this scoping review. Following completion of the review, the protocol and detailed search strategy were retrospectively documented and registered on the Open Science Framework (OSF) to enhance transparency and reproducibility (https://doi.org/10.17605/OSF.IO/DGHXY—accessed on 18 January 2026). The review methodology was predefined and conducted in accordance with the PRISMA-ScR guidelines.

### 2.3. Information Sources and Search Strategy

A systematic search was performed across multiple sources between January 2000 and October 2024. The following categories of information sources were included:

- European and national legislative databases, including EUR-Lex (European Union legislation) and the Romanian legislative portal (legislatie.just.ro) [17];
- Bibliographic databases, including PubMed, Scopus, and Google Scholar;
- Institutional and policy reports from international and European organizations (European Commission, OECD, WHO, EFPIA) [18,19,20];
- National institutional sources, including reports from the National Agency for Medicines and Medical Devices of Romania (NAMMDR) and the National Health Insurance House (CNAS) [21].

Search terms included combinations of: “pharmaceutical legislation”, “medicine access”, “drug availability”, “pricing and reimbursement”, “health technology assessment”, “Romania”, and “European Union”. Reference lists of relevant documents were also screened to identify additional sources.

The search covered the period from January 2000 to October 2024. The final search was run on 15 October 2024 across all sources. For bibliographic databases, we searched PubMed/MEDLINE, Scopus, and Google Scholar. For legislative and institutional sources, we searched EUR-Lex and the Romanian Legislative Portal (legislatie.just.ro), as well as the websites of the European Commission, OECD, WHO, EFPIA, NAMMDR, and CNAS. Database coverage was as follows: PubMed (MEDLINE; 1966–present), Scopus (all years indexed), and Google Scholar (all years indexed). The complete search strategies (including Boolean operators and applied limits) are provided in Appendix A.

The search strategies, limits, and last search dates are reported in sufficient detail to allow for replication of the search process.

In addition to database searches, backward and forward citation tracking of included documents and manual screening of institutional sources were performed to identify further relevant legislative and policy documents not indexed in bibliographic databases. These supplementary strategies were applied systematically to ensure comprehensive coverage of regulatory and grey literature.

### 2.4. Eligibility Criteria

Table 2 shows the eligibility criteria. The time frame from 2000 to 2024 was selected to capture the evolution of modern European pharmaceutical regulation following the adoption of Directive 2001/83/EC and subsequent legislative developments. Documents published in English or Romanian were included, as these languages encompass the vast majority of relevant European and national regulatory and policy sources.

The restriction to English- and Romanian-language publications was based on methodological and pragmatic considerations. English represents the dominant language of peer-reviewed international scientific literature and European institutional reporting, while Romanian covers the full spectrum of national legislative and policy documents directly relevant to the study context. Given the scoping nature of the review—aimed at mapping regulatory and policy frameworks rather than estimating pooled clinical effects—the likelihood that exclusion of publications in other languages would substantially alter the conceptual synthesis was considered low. Nevertheless, we acknowledge that this restriction may have limited the inclusion of certain country-specific or regional analyses published in other European languages. Both peer-reviewed literature and grey literature were included to ensure comprehensive coverage of policy-relevant evidence.

### 2.5. Selection Process

Titles and abstracts were screened independently by two authors. Full-text documents were subsequently assessed for eligibility by the same reviewers. Disagreements were resolved through discussion until a consensus was reached.

To improve methodological rigor, inter-reviewer agreement was assessed during both the title/abstract and full-text screening stages. Percent agreement between reviewers was 92% at title/abstract screening and 95% at full-text review. A pilot calibration exercise was performed on a subset of records to ensure consistent interpretation of eligibility criteria. Discrepancies were addressed through discussion until consensus was achieved. Due to the scoping design and the exploratory objective of mapping regulatory and policy evidence, formal calculation of Cohen’s kappa was not undertaken; however, agreement between reviewers was high following the calibration phase.

### 2.6. Data Charting and Synthesis

A pilot extraction of five randomly selected sources was undertaken to refine the charting form and ensure consistency between reviewers. Data were extracted using a standardized data-charting form developed and piloted by the review team. The form was calibrated on a small subset of included sources prior to full extraction. Data charting was performed independently by two reviewers, and discrepancies were resolved through discussion until consensus was reached. This calibration process reduced the risk of subjective interpretation of policy domains and access indicators. We synthesized the extracted information using a narrative approach and organized findings into pre-specified thematic categories aligned with the review objectives (regulatory harmonization; access barriers; systemic determinants; comparative EU context; implications for vulnerable groups and healthcare utilization). In line with scoping review methodology, no formal critical appraisal of individual sources was undertaken.

The purpose of this review was to map legislative, regulatory, and policy-related evidence rather than to evaluate the effectiveness of specific interventions or generate pooled estimates. Consequently, the emphasis was placed on comprehensiveness of thematic coverage rather than on quality weighting of included studies.

### 2.7. Data Items

For each included source, we charted: (i) bibliographic details (author/institution, year); (ii) source type (EU legislation, national legislation, institutional report, peer-reviewed study, market report); (iii) geographic scope (Romania; EU comparative); (iv) policy domain (authorization/regulation, pricing, reimbursement, HTA, supply/shortages); (v) access indicators reported (timeliness, availability, affordability) and how these were defined in the source; and (vi) the key findings and policy implications relevant to the review objectives.

Together, the reported search strategies, selection procedures, and data-charting approach provide sufficient methodological detail to enable replication of the review process.

## 3. Results

### 3.1. Selection of Sources of Evidence

**Figure 1:**
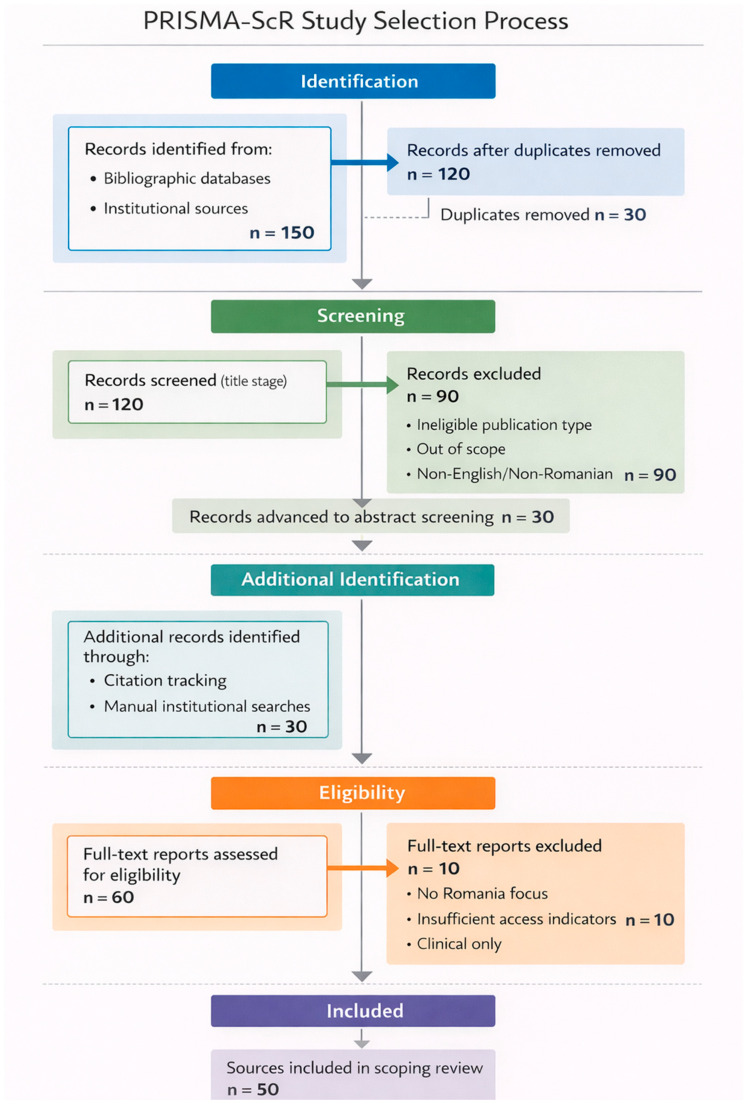
PRISMA-ScR flow diagram of the study selection process. The diagram illustrates the identification, screening, eligibility assessment, and final inclusion of evidence sources in accordance with the PRISMA Extension for Scoping Reviews (PRISMA-ScR) and PRISMA 2020 reporting recommendations. A total of 150 records were identified from bibliographic databases and institutional sources. After removal of duplicates (n = 30), 120 records were screened at title level, and 90 records were excluded. Thirty records advanced to abstract screening. An additional 30 relevant documents were identified through citation tracking and manual institutional searches. In total, 60 full-text reports were assessed for eligibility, of which 10 were excluded due to lack of Romania-specific focus, insufficient policy or regulatory access indicators, or exclusive clinical emphasis without reimbursement implications. Ultimately, 50 sources were included in the scoping review.

A total of 150 records were identified through database and institutional searches. After removal of duplicates, 120 records remained for title screening. Of these, 90 records were excluded for failing to meet the inclusion criteria. The remaining 30 records proceeded to abstract screening. In addition, 30 further relevant documents were identified through backward and forward citation tracking and manual searches of institutional sources. Consequently, a total of 60 documents were assessed for eligibility, resulting in the exclusion of 10 sources as irrelevant to the review objectives or insufficiently policy-relevant. A final total of 50 sources were included in the scoping review. The selection process is illustrated in Figure 1.

At the full-text stage, excluded reports (n = 10) were removed primarily because they did not address Romania directly, did not contain policy-relevant access indicators (timeliness/availability/affordability), or focused exclusively on clinical outcomes without regulatory or reimbursement implications.

### 3.2. Characteristics of Included Sources

The scoping review included a diverse body of evidence comprising European and national legislative documents, institutional and policy reports, and peer-reviewed academic studies published between 2000 and 2024. Included sources addressed pharmaceutical regulation, pricing and reimbursement policies, medicine availability and shortages, and comparative access to medicines across European countries.

Table 3 provides a structured overview of the diversity of included sources, illustrating the predominance of institutional and legislative documents relative to peer-reviewed empirical evaluations. This distribution is important for interpreting the nature of the synthesized evidence, which is primarily policy- and regulation-focused rather than intervention-based.

The scoping review included European and national legislative documents, institutional and policy reports, market analyses, and peer-reviewed academic studies published between 2000 and 2024. No formal critical appraisal of individual sources was performed, in accordance with the objectives and methodology of this scoping review.

The database-specific yields were as follows: PubMed (n = 20 records), Scopus (n = 40 records), and Google Scholar (n = 100 records screened as described in Appendix A), prior to duplicate removal. Google Scholar was included to capture additional grey literature and non-indexed sources; while its search syntax is not fully replicable, we used a structured set of queries with a limited number of results screened for each (i.e., a bounded reproducibility approach) to ensure consistent and transparent retrieval.

Across the included sources, three dominant thematic clusters emerged: (1) structural alignment of Romanian legislation with EU pharmaceutical directives; (2) systemic access barriers driven primarily by national pricing, reimbursement, and supply governance mechanisms; and (3) persistent disparities in comparative EU access indicators, particularly for innovative medicines. Rather than representing isolated findings, these domains collectively illustrate a recurrent pattern: formal regulatory harmonization coexists with substantial functional divergence in access outcomes. A notable geographic imbalance was observed in the included peer-reviewed literature. While institutional and legislative documents were predominantly Romania-specific, much of the comparative empirical research on pricing mechanisms, HTA processes, and parallel trade originated from high-income Western European settings. Evidence directly evaluating the Romanian context remains comparatively limited and often descriptive. This asymmetry reflects broader structural disparities in research production capacity across EU Member States and should be taken into account when interpreting cross-country comparisons.

### 3.3. Legislative Harmonization Between EU and Romanian Pharmaceutical Law

#### 3.3.1. Alignment of Romanian Legislation with EU Directives

Romania’s pharmaceutical regulatory framework is largely built to reflect and comply with European Union legislation. A cornerstone is Directive 2001/83/EC, the EU’s primary code governing medicinal products for human use, which sets rigorous standards for drug authorization procedures, quality, and safety. Romanian law adopted the provisions of this directive (and its updates) through comprehensive national legislation. Notably, Law 95/2006 on healthcare reform incorporated the full spectrum of EU requirements for medicines regulation. Title XVIII of Law 95/2006 addresses all key aspects—from definitions of medicinal products and the criteria for marketing authorization, to manufacturing practice, distribution conditions, labeling, advertising rules, and pharmacovigilance obligations—mirroring the EU standards [12]. This has ensured that, on paper, the processes for approving and monitoring medicines in Romania follow the same principles as in other EU countries.

#### 3.3.2. Adoption of Specific EU Regulations

In addition to general directives, Romania has kept pace with specialized EU pharmaceutical regulations. For example, Regulation (EC) No. 1394/2007 on advanced therapy medicinal products (which covers gene therapies, cell therapies, and tissue-engineered products) was implemented at the national level to govern these complex new treatments [22]. Similarly, updates from Regulation (EC) No. 726/2004 (establishing the European Medicines Agency and centralized EU drug approval) have been reflected in Romanian procedures. The National Agency for Medicines and Medical Devices of Romania (NAMMDR) participates in EU-wide regulatory activities and follows the coordinated procedures for drug approvals and pharmacovigilance established by EU law. Overall, Romanian authorities work within the EU’s single regulatory system for pharmaceuticals, issuing national authorizations or recognizing EU centralized authorizations in line with European Commission decisions.

Synthesizing across thematic domains, the evidence suggests that delays, limited availability, and shortages are not independent phenomena but interrelated consequences of interacting policy levers. Strict external reference pricing, high clawback contributions, delayed HTA procedures, and limited pharmaceutical spending seem to work together, creating a restricted market environment that impacts both manufacturer launch choices and supply consistency. This interaction effect, repeatedly highlighted across institutional and peer-reviewed sources, represents a central explanatory pattern emerging from the mapped evidence.

#### 3.3.3. National Regulatory Framework and Institutions

To operationalize these laws, Romania has put in place numerous secondary regulations and guidelines. Government Decision no. 720/2008, for instance, details the reimbursement categories for medicines (the positive list of drugs covered by the national insurance system) in accordance with EU transparency requirements, and Ministerial Orders regularly update technical norms. Romanian regulatory institutions, primarily the NAMMDR, are charged with ensuring that local practices conform to EU norms. The NAMMDR’s responsibilities include market authorization, inspection of manufacturing and distribution, and post-market surveillance, analogous to those of other EU national competent authorities. The agency also implements harmonized standards in related fields (such as medical devices, through transposed EU directives and regulations) [13].

#### 3.3.4. Gaps in Implementation

Despite this high degree of legal harmonization, some gaps in implementation have been observed. One example is in the area of medication waste collection and disposal. Under EU good practice guidelines, systems should exist for collecting unused or expired pharmaceuticals. While Romanian law assigns NAMMDR the responsibility to ensure such systems are in place, in practice this obligation is not yet fully executed [13]. This indicates that aligning laws with EU directives, while necessary, is not by itself sufficient—adequate infrastructure and enforcement are required to realize the intended benefits. Nonetheless, from a legislative standpoint, Romania has established the fundamental regulatory framework for pharmaceuticals that is broadly consistent with European standards. The following sections examine how this framework translates into outcomes for medicine accessibility.

### 3.4. Barriers to Access to Medicines in Romania

Despite a comprehensive legislative framework, our analysis identified several major barriers that impede the Romanian population’s access to medicines. These obstacles occur at multiple levels—from policy design down to supply and demand dynamics—and help explain the disparity between Romania and other EU countries in timely access to treatment.

**Figure 2:**
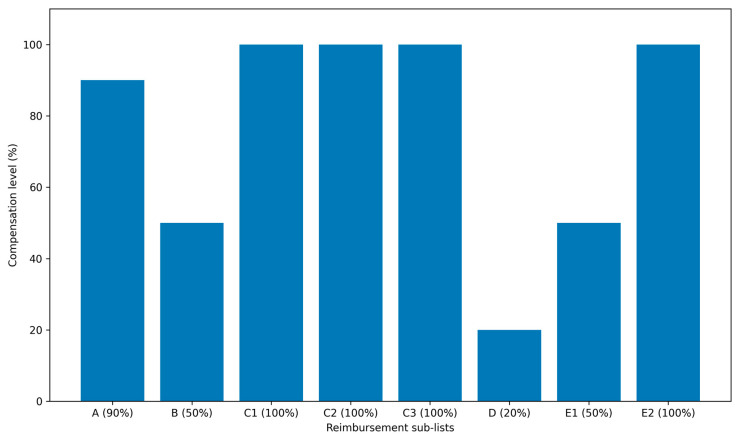
Reimbursement levels by compensation sub-lists in Romania (percentage of reference price or compensation price). The figure illustrates the proportion of the reference price (RP) or compensation price (CP) reimbursed for each reimbursement category (A–E). Source: Romanian Official Gazette; Government Decision No. 720/2008 on reimbursed and free medicines and subsequent amendments. Units: percentage (%). Data extraction date: 10 October 2024. Raw data supporting this figure are provided in Appendix A (Table A1).

Key barriers to medicine accessibility in Romania include:

- Delayed access to new therapies: There are long time lags in making innovative medicines available to patients through reimbursement. On average, Romanian patients wait well over two years, exceeding 800 days for newly approved drugs to become accessible under public insurance. This wait is by far the longest in the EU. By comparison, the average across EU and EEA countries is about 500 days, and in countries like Germany the delay is roughly 120 days. The protracted timeline in Romania is largely due to slow health technology assessment (HTA) and reimbursement decision processes (detailed under systemic factors below). Consequently, many new, effective therapies for diseases such as cancer, autoimmune conditions, or rare diseases are introduced in Romania only after significant delays, if at all. Until the beginning of 2021, only 39 new medicines authorized at the European level had been added to the Romanian reimbursed list—roughly 25% of the total new drugs approved EU-wide in that period [1]. This shortfall means patients must either wait years, pay out-of-pocket for expensive treatments not covered by insurance, or forgo them entirely.
- Limited formulary and high patient co-payments: Romania’s public drug benefit scheme covers only a subset of available medicines, and often only a portion of their cost. The reimbursed drug list is divided into multiple categories (List A, List B, List C, etc.), with each category having a different level of coverage. Many essential or commonly used medicines are on lists that require significant patient co-payment. For example, drugs on List A and B might be only 50–90% reimbursed, with the patient paying the remainder. This leads to a substantial personal cost burden, especially for chronic therapies. If a medication is not included at all in the reimbursement list, patients must pay the full price. Important treatments—even some vaccines (e.g., the varicella/chickenpox vaccine for children)—that are recognized as effective may not be reimbursed, resulting in low demand and intermittent availability in pharmacies [21]. The lack of full coverage for many drugs means that affordability is a major barrier. Low-income patients, in particular, may skip or delay treatment because they cannot afford the co-payment or private purchase. In summary, the structure of the reimbursement system, with its multiple sublists and partial coverage, leaves many patients underinsured for medications, directly limiting access to needed therapies.
- Medicine shortages and discontinuations: An increasingly acute problem in Romania is the shortage of many medicines in pharmacies and hospitals. Over the past decade, interruptions in the medicine supply have escalated, driven by both global and local factors [32]. On the global side, manufacturing disruptions and supply chain issues can affect Romania as they do other countries. Locally, Romania’s market characteristics exacerbate shortages: numerous products have been withdrawn by manufacturers from the Romanian market, often because they are not financially viable underpricing and reimbursement conditions. According to official data from the National Medicines Agency, over 750 medicines for various conditions (including cancer, diabetes, cardiovascular disease, epilepsy, and vaccines) were reported as unavailable or in deficit as of recent listings [28]. These include both innovative drugs and older inexpensive therapies. Patients and healthcare providers frequently struggle to find critical medications, leading to treatment gaps. Shortages force patients to seek alternatives (which may be less effective or tolerable), pay higher prices out-of-pocket for substitutes, or even import medicines personally from abroad. Such scarcity of medicines in Romania represents a significant barrier to continuity of care.
- Low public expenditure on medicines: Underlying many of the above issues is the relatively low level of pharmaceutical spending and healthcare financing in Romania. The country’s expenditure on medicines is about €250 per capita per year, which is well below the Western European average [19]. Although total drug spending has been rising in absolute terms (increasing by roughly 6% annually over the last decade), it remains modest in proportion to need. This low spending reflects strict budget caps on public reimbursement and cost-containment policies. While fiscal prudence keeps drug prices for consumers among the lowest in the EU, it also means fewer resources to encompass new therapies or to stock a broad range of medicines. In 2023, the total value of medicines dispensed to Romanian patients was estimated at 30 billion RON (~€6 billion), which marked a 14% increase from the previous year and about a 60% increase compared to 2012 [20]. Even with this growth Romania’s per capita pharmaceutical expenditure and medicine usage levels trail significantly behind more developed EU healthcare systems. The constrained budget often necessitates tough prioritization—many drugs are left off reimbursement lists or quotas are placed, perpetuating limited access. Figure 1 illustrates the growth in total medicines sales value in Romania over the past decade, reaching ~30 billion RON in 2023.
- Table 4 summarizes the types of patient access to compensated (reimbursed) medicines in Romania’s health system as defined by national policy.
- This multi-tier reimbursement structure, summarized in Table 4 and Figure 2, contributes to substantial patient co-payments and may represent a structural barrier to equitable access to medicines.

As shown in Table 4, only specific categories (C1–C3 and C2 national programs) provide full coverage, while substantial co-payments apply to the majority of outpatient medicines. This structural fragmentation underpins the affordability barriers discussed above.

The structure of outpatient medicine reimbursement in Romania by compensation sub-lists is shown in Figure 2. The figure illustrates the percentage of the reference or compensation price reimbursed for each reimbursement category (A–E), highlighting the fragmentation of the national reimbursement system (Figure 2).

**Figure 3:**
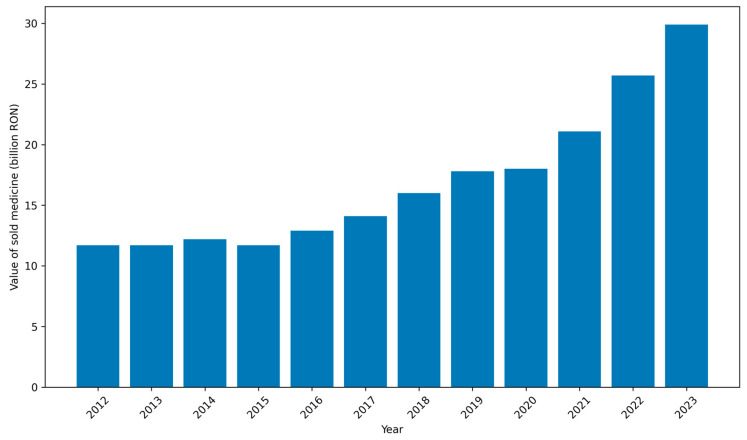
Value of sold medicine in Romania from 2012 to 2023 (in billion Romanian Lei). Source: Statista, Value of sold medicines in Romania (pharmaceutical market sales series). Data extraction date: 12 October 2024. Values include total annual pharmaceutical sales (reimbursed and non-reimbursed medicines) [32]. Aggregated numerical values used to illustrate the market trend in Figure 3 are summarized in Appendix A Table A2. The underlying primary dataset is subject to Statista licensing restrictions and is therefore not publicly shareable.

Overall, these barriers—extensive delays, partial coverage requiring co-payments, frequent drug shortages, and limited health funding—combine to restrict the Romanian population’s access to both innovative and even some basic medicines. The following section examines the systemic and policy factors that underlie these obstacles (Figure 3).

### 3.5. Systemic Factors Contributing to Accessibility Issues

The challenges identified above are rooted in several systemic issues within Romania’s pharmaceutical policy and healthcare system. These factors include regulatory decisions, economic constraints, and administrative processes that collectively affect the availability of medicines. Understanding these systemic causes is crucial for identifying potential solutions. The main contributing factors are:

- Pricing policy and parallel export: The Romanian pricing architecture illustrates a structural paradox: policies designed to maximize affordability at the unit-price level may simultaneously reduce system-level availability by weakening manufacturer incentives and increasing arbitrage dynamics within the EU single market [37]. While this policy aims to make medicines affordable domestically, it has had unintended consequences. Several included sources report that the “lowest price” policy may disincentivize pharmaceutical companies from launching products in Romania, particularly when reference pricing influences broader European price levels [24]. Moreover, the large price differentials have turned Romania into a source for parallel export: intermediaries purchase medicines in Romania at the low regulated price and then export them to higher-price EU markets for profit [26,38]. This practice, legal under EU free market rules, can create or worsen local shortages. As noted by industry experts, Romania’s status as the country with one of the lowest drug price levels in Europe has “allowed the emergence and rapid development of parallel exports, which leads to problems of accessibility to medicines for patients” [3]. In other words, drugs tend to flow out of the Romanian market to where they fetch higher prices, leaving Romanian patients with empty shelves for certain products. Additionally, although manufacturer prices are low, the final cost to Romanian patients can be affected by supply chain mark-ups and taxes. Distribution and pharmacy mark-up rates in Romania are relatively high, and a 9% VAT is applied to medicines (higher than the VAT on medicines in some other EU countries [18]. These add-ons can diminish the affordability gained from low ex-factory prices. Several included sources describe these pricing arrangements as being associated with reduced market incentives and increased supply vulnerability.
- Reimbursement and co-payment system design: Romania’s multi-tiered reimbursement structure (Lists A, B, C, etc.) is a systemic feature that affects access. Many necessary drugs are not fully covered, as discussed in the ‘Barriers’ section. This design is partly a result of budget limits—by requiring co-payments, the health system shifts some costs to patients to control public spending. However, the trade-off is reduced access. Personal contribution (co-payment) for a large portion of medicines is essentially built into the system by design [18]. In other European countries, by contrast, there are caps on out-of-pocket spending, or certain categories of patients (children, elderly, those with chronic diseases) receive medications for free or with minimal fees. Romania’s approach, until now, has been less protective, which is a systemic policy choice. This results in inequities where those who cannot afford the co-pay might not get treatment. The reimbursement lists are updated infrequently and involve a complex approval process (requiring government decisions for additions), which is an administrative rigidity that further delays access to new treatments. Thus, the very structure of the reimbursement mechanism is a contributing factor to limited medicine accessibility.
- Administrative and bureaucratic delays: A significant systemic issue identified is the cumbersome process for approving and funding new medicines in the public system. The timeline from a drug’s European authorization to its inclusion on Romania’s reimbursed list is prolonged by multiple sequential steps: dossier preparation by the manufacturer, health technology assessment by authorities, price setting, budget impact analysis, and finally a government decision to update the reimbursement list. Each step is reported to be lengthy and administratively complex. According to analyses, the main causes of delay include: a significantly long waiting time before companies can even submit reimbursement applications (sometimes due to needing prior inclusion on a special list), a bureaucratic review process with many levels of approval, a restrictive HTA system that may reject drugs not deemed cost-effective enough, and an undersized expert workforce at the national agency handling evaluations [6]. The NAMMDR and Ministry of Health teams responsible for processing reimbursement files are reportedly overburdened and understaffed, slowing down evaluations [19]. Included institutional and academic sources describe administrative and procedural delays as contributing factors to prolonged reimbursement timelines in Romania [6,24]. The requirement that changes to the reimbursement list be formalized through government ordinance is another layer that can introduce months of delay (e.g., waiting for the next government meeting or official gazette publication) [39]. Such systemic administrative barriers are a stark contrast to countries with more agile processes or automatic reimbursement pathways for certain breakthrough drugs.
- Health financing constraints (clawback tax and budget limits): Romania employs a clawback mechanism as a cost-control measure in its pharmaceutical budget. This is a mandatory rebate that drug manufacturers must pay back to the government, calculated as a percentage of their sales to the reimbursed system. The clawback mechanism should be interpreted not merely as a fiscal instrument but as a structural signal of constrained pharmaceutical financing. Its persistence reflects a governance model that prioritizes short-term budgetary predictability over long-term market sustainability. The clawback can render the Romanian market unattractive or even unprofitable for companies, leading them to withdraw products or avoid launching new ones in the country. It is considered a factor in drug shortages and lagging introduction of new molecules. Furthermore, the overall public expenditure on healthcare and medicines is tightly capped—Romania’s healthcare spending as a percentage of GDP is among the lowest in the EU. These financing constraints mean that even when a new drug is deemed important, there may simply not be allocated funds to cover it, or its inclusion might force difficult trade-offs (e.g., delisting another drug or increasing the clawback on industry). The tension between limited resources and the growing cost of innovative therapies is a systemic challenge that underpins Romania’s access issues.
- Supply chain and market structure issues: The pharmaceutical supply chain in Romania has its own structural characteristics that impact medicine accessibility. The distribution network is highly fragmented, with a large number of wholesale distributors and chain and independent pharmacies operating across the country [40]. This can lead to uneven availability, as smaller distributors might not stock low-profit drugs or remote pharmacies may face delays restocking. Moreover, if a few wholesalers export medicines out of Romania, it can quickly drain supply of certain products nationwide. Enforcement of service obligations (requirements for distributors to maintain supply to the domestic market) has historically been weak, which is a systemic regulatory issue. Additionally, Romania relies heavily on imported medicines and raw materials for its pharmaceutical needs—domestic production focuses mostly on generic drugs and is limited in scale. Most active pharmaceutical ingredients (APIs) and many finished products are imported [41]. This dependence on global supply chains means that any international disruption (e.g., manufacturing problems, increased demand elsewhere, or export restrictions by other countries) can directly translate into local shortages. In summary, the combination of a complex multi-actor supply chain and reliance on external sources makes Romania’s medicine supply vulnerable and sometimes slow to respond to patient needs.

**Figure 4:**
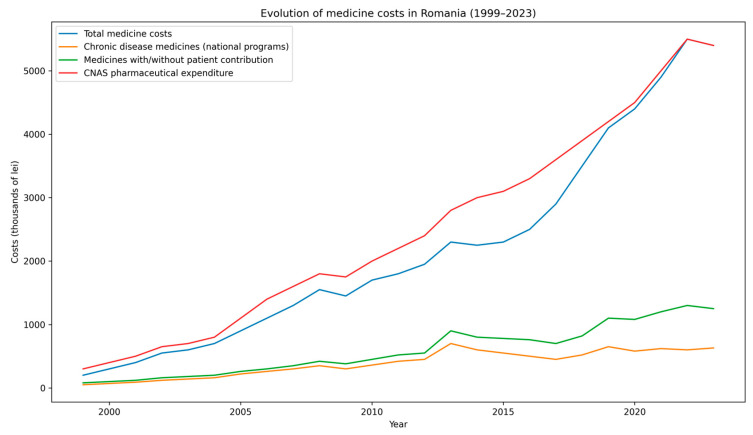
Evolution of medicine-related expenditures in Romania, 1999–2023. Trends in total pharmaceutical expenditure, including reimbursed medicines with and without patient contribution and expenditures within national health programs. Source: National Health Insurance House (CNAS), publicly available annual budget execution and pharmaceutical expenditure reports. Units: thousand Romanian Lei (RON). Data extraction date: 10 October 2024. The figure is author-adapted from CNAS data. Underlying numerical data for Figure 4 are available in Table A3.

Figure 4 shows trends in total pharmaceutical expenditure, costs of medicines with and without patient contribution, expenditure for chronic disease medicines reimbursed through national health programs, and total pharmaceutical spending of the National Health Insurance Fund (CNAS). Source: CNAS [42].

### 3.6. Comparative EU Access Indicators

According to the EFPIA Patients’ W.A.I.T. Indicator 2023–2024, the average time to availability of innovative medicines across EU countries was approximately 578 days, while Romania continued to record one of the longest delays, exceeding 800 days between European authorization and patient access. In terms of availability, fewer than 20% of centrally authorized innovative medicines were available to Romanian patients, compared with an EU average of approximately 46%, based on the latest estimates available at the time of the final search (15 October 2024), with national figures triangulated where possible using public data from NAMMDR and CNAS [8].

In conclusion, Romania’s medicine access challenges are rooted in a confluence of systemic factors: a stringent pricing and reimbursement regime that, while cost-limiting, inadvertently restricts supply and uptake; procedural inefficiencies that delay access; fiscal limitations that constrain what the public health system can provide; and market dynamics that funnel medicines away from patients who need them. These findings set the stage for a broader discussion on how Romania’s situation compares with other countries and what can be done to mitigate these issues.

Detailed data extracted from individual sources, along with their specific contributions to the review objectives, are provided in Appendix A.

## 4. Discussion

This scoping review synthesized evidence from legislative documents, institutional reports, and academic studies to examine how European pharmaceutical regulation and legislative harmonization have influenced access to medicines in Romania. The findings reveal a consistent pattern: despite substantial regulatory alignment with the European Union, Romania continues to experience significant barriers related to timeliness, availability, and affordability of medicines, with important implications for health system performance and equity.

### 4.1. Conceptual Framework: Policy Pathways from Harmonization to Access Outcomes

**Figure 5:**
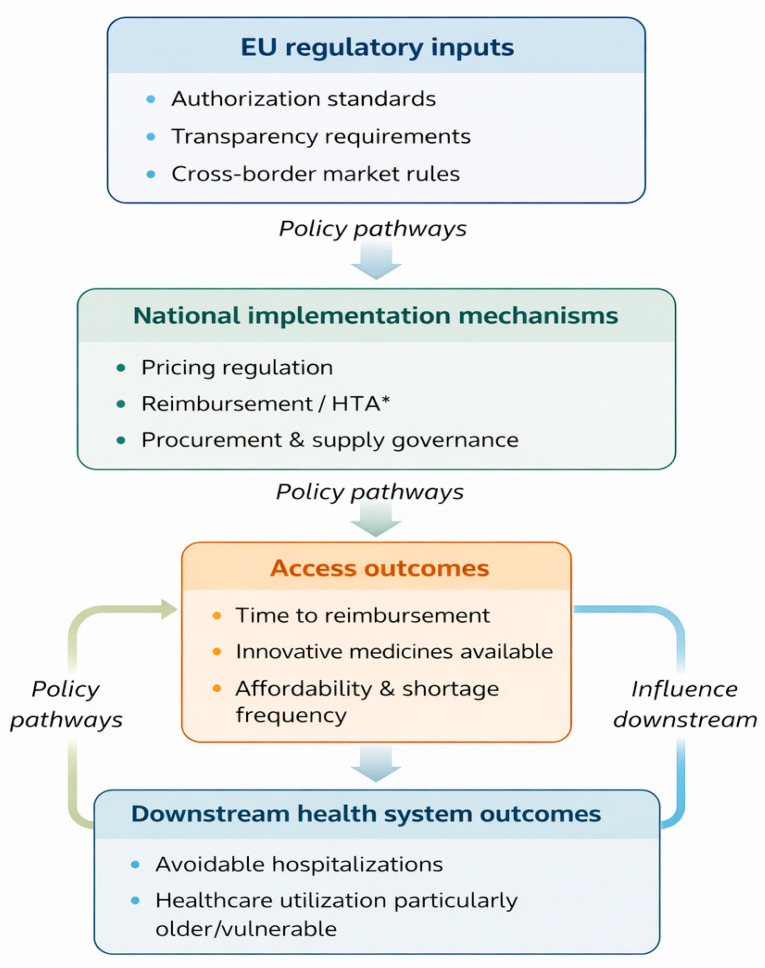
Conceptual framework linking EU pharmaceutical regulation and national policy levers to access outcomes in Romania. EU regulatory inputs interact with national implementation mechanisms (pricing, reimbursement/HTA, and supply governance), shaping access indicators (timeliness, availability, affordability) and downstream health system outcomes. The figure is author-derived based on themes identified in the included evidence. * HTA = Health Technology Assessment, the national evaluation process used to assess the clinical benefit, cost-effectiveness, and budget impact of medicines prior to reimbursement decisions.

To structure interpretation beyond description, we propose a policy pathway framework linking EU legislative harmonization to observed access outcomes in Romania. In this framework, EU regulatory inputs (authorization standards, transparency requirements, and cross-border market rules) interact with national implementation mechanisms (pricing regulation, reimbursement listing procedures, HTA capacity, and procurement/supply governance). These mechanisms shape access outputs (time to reimbursement, proportion of innovative medicines available, affordability and out-of-pocket burden, and frequency of shortages), which in turn influence downstream outcomes such as avoidable hospitalizations and healthcare utilization, particularly among older and vulnerable populations. This framework is summarized in Figure 5.

Comparative access indicators supporting this framework are summarized in Table 5.

Beyond descriptive disparities, the findings illustrate a structural policy trade-off between cost containment and access expansion. Romania’s pharmaceutical governance model prioritizes strict expenditure control through external reference pricing, clawback taxation, and delayed reimbursement listing. While these mechanisms aim to preserve fiscal sustainability, they simultaneously generate market disincentives that affect launch timing, product availability, and supply continuity. This tension reflects broader health policy debates on balancing affordability, sustainability, and equity within constrained public budgets.

As previously noted, the COVID-19 pandemic and geopolitical disruptions acted as stress tests, revealing structural fragilities in national pricing and supply governance rather than creating new ones.

From a broader institutional perspective, Romania’s case exemplifies a pattern of harmonization without convergence within EU pharmaceutical governance. While authorization standards are centralized and legally uniform, pricing, reimbursement, and supply governance remain national competences. This configuration creates asymmetric integration. Legal frameworks are aligned, but real-world access outcomes remain divergent. In this configuration, lower-income Member States face structural constraints that shape manufacturer launch sequencing, reimbursement timelines, and supply stability, reinforcing peripheralization within the internal pharmaceutical market.

This pattern contributes to the broader debate on multi-level pharmaceutical governance in the EU, demonstrating that regulatory convergence at the authorization level does not guarantee distributive convergence in patient access.

### 4.2. Comparison with Other EU Countries

The challenges faced by Romania are not entirely unique—several lower-income EU countries in Central and Eastern Europe grapple with slower introduction of new therapies and periodic drug shortages. However, Romania represents an extreme on many metrics Recent EFPIA Patients’ W.A.I.T. reports were used to update comparative access indicators for the most recent reporting period.

Time to availability refers to the average number of days between European Medicines Agency authorization and patient access through reimbursement or routine availability. Availability of new medicines represents the proportion of centrally authorized innovative medicines available to patients. Values are based on the EFPIA Patients’ W.A.I.T. Indicator 2023–2024 and national HTA reporting. Data reflect the most recent estimates available at the time of the final search (15 October 2024). Percentages and timelines are approximate, as reported by EFPIA and national authorities [43].

Differences are also evident in pricing and reimbursement strategies. Germany’s system involves an independent body (the AMNOG process) that assesses new drugs and sets reimbursement prices based on added benefit, which often results in higher initial prices than in Romania but also ensures availability of those drugs [44]. This contrast reflects a structural policy orientation. While some Western European systems prioritize rapid market entry and broad reimbursement coverage, Romania’s governance model emphasizes expenditure containment, even when this generates secondary effects on launch sequencing and supply stability. Differences in VAT regimes further illustrate divergent policy priorities regarding affordability and fiscal design within the EU.

These findings are consistent with broader European analyses documenting substantial disparities in access to innovative medicines between Western and Eastern European countries, particularly in oncology and other high-cost therapeutic areas [32].

### 4.3. Parallel Trade and EU Regulations

The issue of parallel export of medicines from Romania exemplifies a tension between national healthcare interests and EU single market principles. Parallel trade illustrates a structural tension within EU pharmaceutical governance: national access objectives coexist with single market principles that limit unilateral supply control by lower-priced Member States. The legislative limit here is clear: national authorities are constrained in controlling distribution once the product is in the legitimate supply chain, due to EU trade rules. This calls for an EU-wide approach—something recognized by the European Commission, which in its 2023 proposal highlighted the need to mitigate supply vulnerabilities and ensure better availability in “lower access” markets [44]. Until EU pharmaceutical legislation is updated (potentially introducing obligations for companies to supply all member states or allowing more flexibility to manage shortages), Romania will continue to face challenges stemming from parallel trade and market asymmetries.

From a multi-level governance perspective, Romania’s access challenges highlight the distinction between regulatory convergence and operational convergence. While EU-level legislation harmonizes authorization standards, pricing, reimbursement, and supply governance remain predominantly national competencies. This division creates implementation gaps in which formal legislative alignment does not guarantee equivalent functional outcomes across Member States.

### 4.4. Implications for Healthcare Utilization and Aging Populations

The findings of this review have stark implications. The access barriers and systemic issues in Romania translate directly into worse health outcomes [3].

These access constraints have distributive implications. In systems characterized by substantial co-payments and limited reimbursement breadth, financial barriers disproportionately affect lower-income and elderly populations, potentially amplifying socioeconomic health disparities. Medicine accessibility, therefore, functions not only as a technical reimbursement issue but as a determinant of distributive justice within the health system. Evidence from health systems research shows that inadequate access to timely pharmacological treatment is a major contributor to ambulatory care-sensitive hospitalizations, which are widely used as indicators of inefficient outpatient and pharmaceutical care [45,46].

In rapidly aging societies, such as Romania, persistent barriers to access to essential medicines may undermine health system sustainability by shifting care from preventive and outpatient settings toward more costly inpatient services, a pattern widely observed across European health systems facing demographic aging [47,48].

These challenges are amplified in the context of population aging. Older adults typically require long-term pharmacological therapies and are more vulnerable to interruptions in treatment. In Romania, where demographic aging is accelerating, persistent barriers to medicine access risk intensifying pressure on hospitals and outpatient services. Inefficient pharmaceutical access may therefore undermine the sustainability of the healthcare system by shifting the burden from preventive and outpatient care toward more costly inpatient services.

### 4.5. Legislative Limits and Need for Policy Change

Our analysis shows that having EU-aligned regulations for drug approval and safety, while important, is insufficient to guarantee equitable access. The legislative framework needs to be complemented by proactive national policies that address pricing, reimbursement, and supply chain management. One limitation of the current EU legislative setup is that it leaves pricing and reimbursement almost entirely to member states. This results in a patchwork of 27 systems, where Romania’s system is among the most restrictive. The persistence of the clawback mechanism reflects deeper structural constraints in pharmaceutical financing. Rather than functioning solely as a fiscal correction tool, it signals a system operating under sustained budgetary pressure, with implications for market participation and supply stability.

For example, the EU’s joint procurement mechanisms or collaborative HTA (foreseen under the new EU HTA regulation) could in future help smaller markets like Romania gain better terms or faster access for important therapies.

### 4.6. Comparison of Co-Payment and Coverage

Many EU countries protect vulnerable patient groups from co-payments. In countries like the UK, out-of-pocket costs for prescriptions are capped or waived for children, the elderly, and low-income patients; in France and Germany, most essential drugs for serious conditions are fully reimbursed. Romania’s policy of broad co-payments on lists A and B stands out as more burdensome. This suggests Romania could look to its peers for models to reduce financial barriers—for instance, increasing the proportion of fully reimbursed medications for chronic and life-threatening illnesses, or implementing an annual cap on patient drug expenses. The implication is that legislative harmonization must extend beyond technical regulatory matters to the realm of health financing and insurance design, which is politically and economically challenging.

### 4.7. Ongoing Reforms and Future Prospects

The situation in Romania is dynamic, and there have been some recent positive developments. Notably, towards the end of 2020 and in 2021, the government added a significant number of new molecules (approximately 70) to the reimbursement list [48]—an unprecedented update that somewhat improved the availability of novel treatments. This was likely driven by both internal pressure and external influence (e.g., the EU’s emphasis on equitable access in light of the COVID-19 experience). Additionally, legislative updates such as Law 289/2023 (passed in 2023) indicate ongoing efforts to adjust the legal framework—though the content of that law pertains to specific aspects (it may amend pricing or other regulations), it reflects an awareness at the legislative level of the need for change [19]. The European Commission’s pharmaceutical strategy, if implemented, could shorten authorization-to-access times by incentivizing companies to launch products in all member states (through regulation of market exclusivity incentives). Romania stands to benefit from such measures, but they are still in proposal stages.

At the national level, potential reforms include revising external reference pricing, adjusting the clawback mechanism to enhance market sustainability, streamlining HTA and reimbursement procedures, and strengthening supply chain governance. Implementing these measures would require targeted legislative changes and coordinated action among policymakers, industry stakeholders, and European partners.

Table 6 summarizes key EU- and Romania-level policy milestones that are likely to shape pricing, reimbursement timelines, and supply stability. The timeline is provided to contextualize the access indicators discussed in this scoping review and to support interpretation of observed patterns; it is not intended to imply causal attribution.

### 4.8. Limitations of This Review

This scoping review has several limitations. First, consistent with scoping review methodology, we did not conduct a formal critical appraisal of included sources. As a result, the synthesis does not differentiate findings based on methodological quality, risk of bias, or strength of evidence. While this approach is appropriate for exploratory mapping of heterogeneous regulatory and policy documents, it limits the ability to draw graded conclusions regarding causal relationships between specific policy levers and access outcomes. The proposed conceptual framework should therefore be interpreted as an evidence-informed synthesis of reported patterns rather than a hierarchy-weighted evaluation of policy effectiveness. Second, although we used multiple databases and targeted institutional sources, Google Scholar results are partly influenced by relevance algorithms and may not be perfectly reproducible; to mitigate this, we reported the exact queries used and screened a predefined number of results per query. Third, access and shortage indicators can change rapidly and are periodically updated by national and European institutions; consequently, some quantitative estimates may evolve after the final search date (15 October 2024).

Moreover, because no formal critical appraisal was conducted, the Discussion section emphasizes structural interpretation rather than graded evaluation of evidence strength. The proposed policy pathway framework should therefore be interpreted as an evidence-informed conceptual synthesis rather than a hierarchy-weighted assessment of causal effectiveness.

Fourth, we restricted inclusion to documents published in English or Romanian. Both feasibility and conceptual considerations guided this decision. English is the primary language of international academic publishing and of European institutional documentation, including reports from the European Commission, the OECD, the WHO, and EFPIA. Romanian was essential for capturing national legislation, governmental decisions, and institutional reports that were not systematically available in other languages.

An additional limitation relates to the geographic distribution of the evidence base. Much of the empirical literature on pharmaceutical pricing, HTA efficiency, and market dynamics originates in high-income Western European countries, where institutional capacity, funding levels, and health system design differ substantially from those in Romania. Consequently, extrapolation of certain mechanisms to the Romanian context may require cautious interpretation. The relative scarcity of Romania-specific empirical evaluations underscores the need for locally grounded quantitative and policy implementation research.

As the objective of this scoping review was to map regulatory and policy frameworks rather than to estimate pooled quantitative effects, we considered that excluding non-English and non-Romanian publications was unlikely to materially affect the conceptual analysis of legislative harmonization and access pathways. However, it remains possible that relevant comparative analyses or policy discussions published in other European languages (e.g., French, German, Spanish, or Italian) were not captured. Therefore, the findings should be interpreted with awareness of this language restriction.

Finally, because this review maps policy and regulatory evidence, it cannot establish causal inference between specific policy levers and health outcomes. These limitations may contribute to the relative overrepresentation of institutional perspectives and underrepresentation of independent empirical evaluations, potentially affecting the balance of the synthesized evidence.

At the European level, the findings suggest that legislative harmonization without coordinated alignment of pricing and reimbursement incentives may unintentionally perpetuate intra-EU inequalities. Future reforms under the European pharmaceutical strategy may need to incorporate mechanisms to reduce the peripheralization of lower-income markets and to promote simultaneous, equitable launch strategies across Member States.

## 5. Conclusions

This scoping review reveals that Romania’s population continues to face significant impediments in access to medicines, despite the country’s formal compliance with European pharmaceutical regulations. The critical examination of laws, policies, and outcomes highlights a disconnect between legislative harmonization and actual patient access. In summary, while EU and national regulations ensure that medicines in Romania meet high standards of quality and safety, they have not yet ensured the timely or equitable availability of those medicines to all who need them.

Several key conclusions and actionable recommendations emerge from our analysis:

- Bridging the access gap requires policy shifts: The root causes of poor medicine accessibility in Romania lie in policy choices regarding pricing, reimbursement, and health financing. To align patient access with that of other EU countries, Romania will need to adjust these policies. Notably, it would be beneficial to eliminate the current high co-payments for essential drugs. This could be achieved by doing away with the multi-tier reimbursed drug lists (A, B, E) that require patient contributions and instituting a single comprehensive medicines list fully covered (100% reimbursement) by the National Health Insurance House [4]. Such a reform would ensure that inexpensive but vital medications are free at the point of care for all patients, removing a major financial barrier.
- Increase funding and streamline processes for new medicines: To reduce the long lag in new therapy availability, Romania should allocate greater budgetary resources toward innovative medicines and modernize its reimbursement approval process. This means not only increasing the pharmaceuticals budget (recognizing that spending on effective treatments is an investment in population health), but also simplifying bureaucratic procedures. Establishing clearer timelines and accountability for each step of the HTA and listing process, possibly integrating EMA decisions more directly, could shorten the time to reimbursement. Enhancing the capacity of national agencies (through the hiring of additional experts and improved funding for HTA processes) is equally important so that Romania can evaluate and adopt innovations without undue delay.
- Reassess pricing and market policies to ensure supply: A re-evaluation of the current pricing and rebate architecture is necessary to restore market sustainability and reduce structural disincentives affecting launch timing and supply continuity.
- Romania may consider adopting a balanced pricing strategy—for example, using average European prices or engaging in negotiated managed-entry agreements for costly novel drugs—to make participation in the market more attractive to companies while still ensuring affordability for the health system. Additionally, targeted policy tools to combat excessive parallel export should be used: stronger monitoring of distributors, swift export restriction mechanisms for medicines in short supply, and collaboration with EU authorities to develop regional solutions. Ensuring a stable supply of medicines might also involve maintaining strategic stocks of critical medications and encouraging local production of drugs prone to shortages.
- Leverage European collaboration and upcoming legislative reforms: Romania should actively engage with and leverage the upcoming EU pharmaceutical reforms that aim to reduce inequalities in access. This includes supporting proposals that reward or mandate broader availability of medicines across all member states, participating in joint procurement or pricing negotiations for high-cost therapies (as has been done for vaccines), and utilizing the new EU-wide HTA cooperation to inform national decisions. By being a vocal stakeholder at the EU level, Romania can help shape policies that will benefit smaller markets. Domestically, aligning any new national legislation (such as amendments in 2023 and beyond) and improving access, rather than solely focusing on cost containment, will be crucial.
- Continuous monitoring and stakeholder engagement: Finally, improving medicine accessibility should be seen as an ongoing commitment. The government, together with patient organizations, healthcare professionals, and industry representatives, needs to establish a continuous monitoring mechanism for access to medicines. Regular analysis of indicators (such as the number of new drugs introduced, stock-out frequencies, patient out-of-pocket spending, etc.) can guide timely interventions. Stakeholder engagement is essential: involving patient advocacy groups in decision-making can help ensure that policies remain patient-centered and that proposed solutions (such as removing copayments or adjusting prices) effectively address the community’s needs.

In conclusion, addressing the accessibility of medicines in Romania will require a multifaceted strategy that combines legislative action, policy reform, and investment. The findings of this review strongly suggest that without such concerted efforts, Romania will continue to lag behind its European counterparts in providing life-saving and life-improving treatments to its population. Conversely, by implementing the recommended changes—easing financial barriers, expediting access to innovation, and securing the drug supply—Romania can move towards a more equitable healthcare system where regulatory harmonization finally translates into real-world health benefits. These steps are not only critical for Romania’s patients but also align with the broader European vision of “fair access for all” in healthcare. The experience of Romania thus serves as both a cautionary tale and a call to action: robust pharmaceutical regulation must be matched by robust measures to ensure that medicines reach the people who need them.

## Figures and Tables

**Table 1:** Key objectives and focus areas.

Objective	Description
Regulatory framework mapping	To map the European pharmaceutical regulatory framework and its transposition into Romanian legislation
Barriers to access to medicines	To identify and synthesize evidence on barriers to access to medicines in Romania, including timeliness, availability, and affordability
Comparative analysis	To compare access to medicines in Romania with that of other European Union Member States
Healthcare and population impact	To explore the implications of pharmaceutical access barriers for healthcare utilization and vulnerable populations

**Table 2:** Criteria.

Category	Eligibility Criteria
Scope of content	Documents addressing pharmaceutical legislation, pricing, reimbursement, or access to medicines
Geographic relevance	Documents referring to Romania or providing comparative European data relevant to the Romanian context
Language	Documents published in English or Romanian
Publication period	Documents issued between 2000 and 2024
Exclusion criteria	Documents focusing exclusively on clinical efficacy or biomedical outcomes without policy, regulatory, or access-related implications

**Table 3:** Characteristics of sources included in the scoping review.

Source (Author/Institution, Year)	Type of Source	Geographic Scope	Main Topic(s) Addressed	Relevance to Review Objectives
European Commission (2023) [3]	Institutional policy report	European Union	Pharmaceutical legislative reform, access disparities	Provides EU-level policy context and reform proposals addressing unequal access across Member States
WHO (2004) [4]	International policy framework	Global	Access to medicines	Conceptual framework for availability and affordability
Opriș et al. (2023) [5]	Peer-reviewed study	Romania	Pharmaceutical market strategies	Explains industry response to national policies
OECD (2018) [7]	International institutional report	International/EU	Pharmaceutical innovation and access	Comparative data on spending and access across health systems
Law No. 95/2006 (Romania) [10]	National legislation	Romania	Healthcare reform, medicines regulation	Main national legal act transposing EU pharmaceutical directives
Directive 2001/83/EC (2001) [13]	EU legislation	European Union	Medicinal product regulation	Core legislative framework transposed into Romanian law governing authorization and safety
OECD/EC (2024)—EU Country Cancer Profile [21]	Institutional report	Romania/EU	Access to oncology medicines, outcomes	Highlights disparities in access to high-cost innovative therapies
Regulation (EC) No. 1394/2007 (2007) [22]	EU legislation	European Union	Advanced therapy medicinal products	Illustrates adoption of specialized EU pharmaceutical regulation in Romania
Government Decision No. 720/2008 (Romania) [23]	National legislation	Romania	Reimbursement lists and compensation categories	Defines national reimbursement structure affecting affordability
Kanavos et al. (2017) [24]	Peer-reviewed study	Europe	External reference pricing, reimbursement	Explains systemic effects of pricing policies relevant to Romania
Uyl-De Groot et al. (2020) [25]	Peer-reviewed study	Europe	Access to cancer medicines	Comparative evidence of cross-country variation in access to newly registered cancer medicines across Europe
Kyle (2011) [26]	Peer-reviewed study	Europe	Parallel trade	Explains impact of parallel export on medicine availability
Focșa et al. (2022) [27]	Peer-reviewed study	Romania	Medicine shortages	Documents real-world impact of shortages on practice
Taerel & Țurcu (2009) [28]	Peer-reviewed study	Romania	Range of authorized medicines	Early evidence of limited national pharmaceutical availability
SeeNews (2012) [29]	Market analysis report	Romania	Pharmaceutical market structure	Contextualizes market dynamics and investment climate
GlobalData (2021) [30]	Market analysis report	Romania	Healthcare and pharmaceutical system	Provides economic and policy background
Euromonitor International (2021) [31]	Market research report	Romania	Pharmaceuticals market trends	Supports analysis of supply and demand constraints
Statista (2024) [32]	Statistical database	Romania	Pharmaceutical expenditure	Provides longitudinal expenditure data
CNAS (2024) [33]	National institutional report	Romania	Health insurance budget	Context for financing constraints
Panteli et al. (2016) [34]	WHO Europe report	Europe	Pharmaceutical regulation	Comparative regulatory approaches
Toma & Crișan (2021) [35]	Peer-reviewed comparative study	Europe	Regulatory differences	Shows diversity of national regulatory implementation
Al-Worafi (2020) [36]	Book chapter	Global	Drug regulation	Broader regulatory perspective relevant to emerging markets

**Table 4:** Types of patient access to compensated medicines in Romania.

Sub-List	Description (According to National Reimbursement Legislation)	Reimbursement Basis	Reimbursement Level (%)	Patient Contribution
A	International Non-proprietary Names (INNs) reimbursed for outpatient treatment under the basic reimbursement scheme	Reference Price (RP)	90%	Reference price + co-payment
B	INNs reimbursed for outpatient treatment with partial coverage	Reference Price (RP)	50%	Reference price + co-payment
C1	INNs reimbursed at full coverage for specific disease groups in outpatient care	Reference Price (RP)	100%	Reference price
C2	INNs included in national health programs, reimbursed in ambulatory and hospital settings	Compensation Price (CP)	100%	Compensation price
C3	INNs reimbursed at full coverage for children (≤18 years), young adults (18–26 years in education without income), pregnant women and women with newborns	Reference Price (RP)	100%	Reference price
D	INNs reimbursed for outpatient treatment with minimal public coverage	Reference Price (RP)	20%	Reference price + co-payment
E1	Immunological INNs for active immunization reimbursed for selected population groups	Reference Price (RP)	50%	Reference price + co-payment
E2	Immunological INNs for active immunization reimbursed for selected population groups	Reference Price (RP)	100%	Reference price

Notes: RP = reference price; CP = compensation price. Source: Romanian Official Gazette; national reimbursement legislation, Government Decision No. 720/2008 on reimbursed and free medicines and subsequent amendments [21]. Data extraction date: 10 October 2024.

**Table 5:** Comparative access to innovative medicines in Romania, the EU average, and Germany.

Country	Time to Availability (Days)	Availability of New Medicines (%)	Source
Romania	>800	<20	EFPIA W.A.I.T. 2023–24
EU average	~578	~46	EFPIA W.A.I.T. 2023–24
Germany	~120	>80	EFPIA/national HTA

**Table 6:** Timeline of key policy and regulatory milestones relevant to access to medicines in Romania.

Year	Policy/Regulatory Change	Expected Mechanism (How It Can Affect Access)	Data Source (Cite)
2014	Romania: Ministerial Order No. 861/2014 (HTA criteria/scorecard model for inclusion of medicines in reimbursement)	Formalizes HTA evaluation criteria for medicines; can influence time-to-reimbursement and listing decisions, affecting timeliness and availability of reimbursed medicines.	Romanian medicines authority (ANMDMR)—Order 861/2014.
2020 (May)	Romania: Law 53/2020 introducing differentiated clawback contributions (innovative vs. generic)	Differentiated clawback aims to improve market sustainability for certain products; may affect launch/withdrawal decisions, influencing availability and potentially shortages.	CMS Law-Now summary; also reported by business/industry policy sources.
2023 (April–May)	Romania: revision of clawback policy (cap/differentiated rates), including 25% for innovative and 15% for generics	Lower effective burden for generics can reduce withdrawals and support supply continuity; differentiated clawback may reduce shortage risk and improve availability for low-margin products.	CNAS official communication (May 2023); corroborated by US State Dept Investment Climate Statement.
2023 (April)	EU: European Commission pharmaceutical legislation reform package (Communication COM/2023/190 and legislative proposals COM/2023/192)	Intended to improve timely and equitable access across Member States; may introduce incentives/requirements affecting launch timing and supply obligations, potentially narrowing East–West access gaps.	EUR-Lex: COM/2023/190 final and proposal COM/2023/192 final.
2021–2025	EU: Regulation (EU) 2021/2282 on Health Technology Assessment (HTAR)—entered into force in January 2022, applies from 12 January 2025; first scope includes oncology medicines with new active substances and ATMPs	Introduces EU-level joint clinical assessments (JCAs); may streamline national HTA inputs and reduce duplication, with potential effects on reimbursement timelines and timeliness of access (depending on national implementation).	EUR-Lex Regulation text (entry into force + date of application); European Commission HTA implementation pages; EMA notice on applicability from 12 January 2025.
2024	Romania: reduced VAT rate of 9% applies to medicines for human/veterinary use	Lower VAT reduces end-user price and can improve affordability; however, pricing and reimbursement design may still drive access barriers.	US Department of Commerce Country Commercial Guide (January 2024).
2025 (August)	Romania: VAT reform package—standard VAT increases (19%→21%) and reduced VAT rates consolidated into a single 11% rate including medicines	VAT shift can affect affordability (patient out-of-pocket price) and potentially demand/supply dynamics; should be considered when interpreting post-2025 access indicators.	EY Tax Alert and other tax compliance summaries.

## Data Availability

No new data were created or analyzed in this study. Data sharing does not apply to this article. Data underlying Figure 2 and Figure 4 are derived from publicly available Romanian legislative and CNAS sources and are provided as raw datasets in the Appendix A (Table A1 and Table A3). The data underlying Figure 3 are based on published Statista market summaries and are subject to licensing restrictions; therefore, only aggregated values used for illustrative trend analysis are reported.

## Supplementary Materials

The following supporting information can be downloaded at https://www.mdpi.com/article/10.3390/healthcare14050688/s1, Preferred Reporting Items for Systematic reviews and Meta-Analyses extension for Scoping Reviews (PRISMA-ScR) Checklist.

## Abbreviations

The following abbreviations are used in this manuscript:

CNAS	National Health Insurance House (Romania)
EMA	European Medicines Agency
EU	European Union
HTA	Health Technology Assessment
INN	International Non-proprietary Name
NAMMDR	National Agency for Medicines and Medical Devices of Romania
OECD	Organisation for Economic Co-operation and Development
WHO	World Health Organization

## Appendix A

### Appendix A.1. PubMed (MEDLINE) Search Strategies (–Last Run: 15 October 2024)

**Search Fields**	**Query**	**Limits Applied**
Title/Abstract	((“pharmaceutical legislation” [Title/Abstract] OR “pharmaceutical regulation” [Title/Abstract] OR “drug policy” [Title/Abstract] OR “pricing policy” [Title/Abstract] OR “reimbursement” [Title/Abstract] OR “health technology assessment” [Title/Abstract] OR HTA [Title/Abstract] OR “medicine shortage*” [Title/Abstract] OR “drug shortage*” [Title/Abstract] OR “parallel export*” [Title/Abstract]) AND (“access to medicine*” [Title/Abstract] OR “access to medicines” [Title/Abstract] OR “drug availability” [Title/Abstract] OR “medicine availability” [Title/Abstract] OR affordability [Title/Abstract] OR “time to reimbursement” [Title/Abstract] OR “time to access” [Title/Abstract]) AND (Romania [Title/Abstract] OR Romanian [Title/Abstract]) AND (“European Union” [Title/Abstract] OR EU [Title/Abstract] OR Europe [Title/Abstract] OR “European legislation” [Title/Abstract]))	publication years 2000–2024; language English OR Romanian

### Appendix A.2. Scopus—Last Run: 15 October 2024

**Search Fields**	**Query**	**Limits Applied**
Title-Abstract-Keywords	TITLE-ABS-KEY ((“pharmaceutical legislation” OR “pharmaceutical regulation” OR “drug policy” OR reimbursement OR pricing OR “health technology assessment” OR HTA OR “medicine shortage” OR “drug shortage*” OR “parallel export*”) AND (“access to medicine*” OR “access to medicines” OR “drug availability” OR “medicine availability” OR affordability OR “time to reimbursement” OR “time to access”) AND (Romania OR Romanian) AND (“European Union” OR EU OR Europe OR “European legislation”))	publication years 2000–2024; document types: articles, reviews, reports, book chapters (where relevant).

### Appendix A.3. Google Scholar—Last Run: 15 October 2024

Google Scholar does not support a fully identical Boolean syntax to that of bibliographic databases; therefore, we used a structured set of queries and consistently screened results. We ran the following queries and screened a predefined bounded subset of results (total n = 100 across queries, sorted by relevance, applying the year filter (2000–2024) and language (English/Romanian) where available:

1. Romania “access to medicines” European Union reimbursement pricing
2. Romania pharmaceutical legislation harmonization, EU reimbursement
3. Romania “medicine shortages” pricing policy “parallel exports”
4. Romania “health technology assessment” reimbursement delays medicines

We also screened the reference lists of the included key documents to identify additional eligible sources.

**Table A1:** Reimbursement levels by compensation sub-lists in Romania.

Reimbursement Sub-List	Description (According to National Legislation)	Reimbursement Basis	Reimbursement Level (%)	Patient Contribution
A	International Non-proprietary Names (INNs) reimbursed for outpatient treatment	Reference Price (RP)	90%	Reference price + co-payment
B	INNs reimbursed for outpatient treatment with partial coverage	Reference Price (RP)	50%	Reference price + co-payment
C1	INNs reimbursed for specific disease groups in outpatient care	Reference Price (RP)	100%	Reference price
C2	INNs included in national health programs (ambulatory and hospital care)	Compensation Price (CP)	100%	Compensation price
C3	INNs reimbursed for children (≤18 years), young adults (18–26 years in education without income), pregnant women, and women with newborns	Reference Price (RP)	100%	Reference price
D	INNs reimbursed for outpatient treatment with minimal coverage	Reference Price (RP)	20%	Reference price + co-payment
E1	Immunological INNs for active immunization for selected population groups	Reference Price (RP)	50%	Reference price + co-payment
E2	Immunological INNs for active immunization for selected population groups	Reference Price (RP)	100%	Reference price

Source: Romanian Official Gazette; Government Decision No. 720/2008 on reimbursed and free medicines and subsequent amendments. Data extraction date: 10 October 2024. Units: percentage (%).

Table A1 provides the raw reimbursement percentages by compensation sub-list used to generate Figure 2. Values were extracted from Government Decision No. 720/2008 and subsequent amendments published in the Romanian Official Gazette.

**Table A2:** Summary of published market estimates for the value of medicines sold in Romania.

Year	Value of Medicines Sold (Billion RON)
2012	18.8
2013	19.3
2014	20.1
2015	21.4
2016	22.6
2017	23.8
2018	24.9
2019	26.1
2020	26.8
2021	27.9
2022	29.3
2023	30.0

Note: Values are reported as aggregated estimates summarized from publicly available Statista market overviews. The table is provided for visual trend interpretation only and does not represent the primary raw data. Licensing conditions restrict access to the original Statista dataset.

**Table A3:** Pharmaceutical expenditure in Romania by category (1999–2023).

Year	Total Pharmaceutical Expenditure	Reimbursed Medicines (with Patient Contribution)	Reimbursed Medicines (Without Patient Contribution)	National Health Programs (Chronic Diseases)
1999	1,245,000	734,000	211,000	300,000
2003	2,110,000	1,205,000	415,000	490,000
2007	4,380,000	2,480,000	980,000	920,000
2010	6,950,000	3,870,000	1,540,000	1,540,000
2013	10,320,000	5,680,000	2,340,000	2,300,000
2016	14,250,000	7,720,000	3,250,000	3,280,000
2019	18,900,000	9,860,000	4,380,000	4,660,000
2021	23,500,000	12,240,000	5,680,000	5,580,000
2023	30,000,000	15,600,000	7,200,000	7,200,000

Values are aggregated from annual CNAS financial execution reports. Categories reflect the CNAS reporting structure at the time of data extraction. Used to generate Figure 4. Source: National Health Insurance House (CNAS), publicly available budget and expenditure reports. Units: thousand Romanian Lei (RON). Data extraction date: 10 October 2024.
